# A Treatise on Medical Study, or on the Method of Studying and of Teaching Medicine

**Published:** 1840-07

**Authors:** 


### Art. VII.

*Traité des Etudes Médicales, ou de la Manière d'étudier et d'enseigner la Médecine.* Par E. Fred. Dubois (d'Amiens), Professeur agrégé à la Faculté de Médecine de Paris, &c. &c.—*Bruxelles,* 1838. 12mo, pp. 460.

*A Treatise on Medical Study, or on the Method of Studying and of Teaching Medicine.* By E. Fred. Dubois (of Amiens.)

*Observations on Medical Education, with a view to Legislative Interference.* By Richard Jones, m.r.c.s. &c.—*London,* 1839. 8vo, pp. 52.

In pursuance of the plan commenced in our last Number, we shall now proceed to lay down what we conceive to be the *Principles of Medical Education.* It is not our intention to discuss in detail the best method of adapting the various systems at present in operation to our own standard. This may be done better hereafter. But, whatever legislative changes are proposed, we deem it important that they should be rightly directed. We shall proceed, therefore, as if we had a medical faculty to create; and we shall build our system of education upon these two foundations:—the nature, objects, and connexions of the different departments of medical knowledge considered in the abstract; and the capacity of the average of well-educated young men for the acquirement of these.

Commencing from the point at which we left off in our former article, we shall suppose a youth committed to our care who had undergone the training there recommended; and that we were desired to fit him in the best manner we were able for the future practice of medicine or surgery, without regard to existing institutions. Now, if our formerly-expressed ideas be tolerably correct, our pupil will commence the study of his profession with the following advantages:—He will have acquired habits of close and accurate observation. He will have learned not to trust too implicitly to what is commonly termed the evidence of his senses; whilst he never allows those senses to remain inactive. He will have acquired, also, the tendency to reason upon the phenomena which he observes; to bring the principles with which he is acquainted to bear upon them; and to seek for new principles by which to explain what he cannot comprehend in his previous generalizations. He will have been accustomed, moreover, to deduce results from principles, and thus to *expect* occurrences, which will or will not verify his predictions, according as his reasoning has been correct or fallacious. Further, he will have had some practice in that most difficult, and to the medical man most important art—the analysis of complex phenomena; and he will have seen the mode in which a number of causes may unite to produce an effect which in itself appears simple. He will have acquired something of that *mental tact* which discovers the fallacy of reasoning that appears rigid and conclusive; and which, on the other hand, allows their due weight to evidence and arguments which cannot be termed demonstrative. And, above all, he will have had a glimpse of the beauty and majesty of truth; and will have perceived the necessity for the absolute dismissal of prejudice in the search for it.

We have argued upon abstract grounds that education should proceed from the *general* to the *special;* and that, up to a certain point, the course pursued should be nearly the same in all instances. When the subsequent occupation of the mind has been determined on, it will be time to direct it in that peculiar channel. But we think the early choice of a profession, before the mental powers have had time to develope themselves, and the tastes have been in some degree displayed, a very injurious plan. Of course there will be exceptions, arising out of peculiar external circumstances, or of some very decided bent on the part of the individual; but we are speaking only of general principles. Too often does it happen that youths are placed, with scarcely any choice of their own, or perhaps even in opposition to their wishes, in a business or profession for which they have no inclination or even a decided disgust. We are all accustomed to condemn the too early choice of a wife, and still more to reprobate those *mariages de convenance* which result from interested motives on the part of the parent, and in which the hearts of the individuals concerned are not taken into the account. Now we would ask, is the choice of a wife a matter of such immensely superior importance to the choice of the employment to which the whole subsequent life is to be dedicated—which is to be its business, and should as much as possible be its pleasure—that the one must be deferred until the individual has attained years of discretion, and must then be left principally to himself, whilst the other is decided upon when his mind is altogether immature, his higher capabilities scarcely manifesting themselves, and his own judgment so little to be depended upon, that it is almost entirely omitted from the calculation? It will, we think, be generally acknowledged that *he* has the best prospect of success in his profession or employment whose heart as well as his head is engaged in it; in other words, who pursues it as a favorite object, not merely as his means of livelihood. How often do we see men *obliged* to devote their intellectual powers to some occupation laborious and distasteful to themselves, yet by others relished as the constant happiness of life. And do such men succeed? The earnestness with which they prepare themselves for what they consider their duties, and the steady conscientiousness which governs their discharge of them, must give them a certain measure of success; but that measure is far below their deserts. This is peculiarly the case in medicine. How frequently does a comparatively superficial practitioner rapidly acquire a lucrative practice; while the man possessed of ten times his professional merits struggles with neglect and poverty. The world is very quick in discovering the real tastes of those who are candidates for public confidence; and, nine times out of ten, he will be preferred who *shows* that he likes his profession to him who has perhaps a higher intellectual pleasure in the pursuit of it, but whose taste cannot avoid being offended with its details. Mr. A. is known to enjoy the quiet solitude of his study, and to occupy his leisure hours in the pursuit of literary or scientific knowledge; Mr. B. is a favorite at dinner parties and balls, and an amusing gossip in the sick room. Of Mr. A. the public will say "clever man, sure to get on;" but so he will remain to the end of his days, never *getting on* beyond a certain point; whilst the patients and their fees go to Mr. B., because he makes it evident that he likes nothing better than practice.

This may, perhaps, be considered rather an overdrawn picture; but many of our readers, we doubt not, will acknowledge its general fidelity. Our object in introducing it has been simply to illustrate our position that the *taste* as well as the *intellect* must be consulted in the choice of a profession, and especially in the decision which renders medicine the object of future pursuit. If the taste is strong it will lead to the successful employment of the intellect upon the objects towards which it is directed; if it be deficient, the chance of success from the forced application of the mental powers is very small. It is very seldom that, in boys of fifteen or sixteen years old, the nicer shades of either mental or moral character can be discriminated. Indeed, this is the time when both are undergoing the most important changes. To a certain degree these changes may be directed and controlled, but only within particular limits; and it seems to us a great error to determine upon a profession at this *transition epoch* of the mental history. Now, if such a course of general education as we have been recommending be prolonged for two or three years, say, as an average, to the age of eighteen, the mind will have had time to unfold itself; its powers are ready to be employed upon whatever is chosen as the special object of pursuit with full intensity; and, a greater variety of these objects having been presented to the student's attention, he will be able to make a choice among them with less ignorance of their conformity to his tastes and habits of thought than when he merely contemplates them, as it were, from a distance.

Among the most prejudicial errors of the present system of academical instruction, we reckon the large share of time and attention bestowed upon classical study. We have already recorded our opinion of its value as a *means* of intellectual discipline; and our entire agreement with the propriety of the regulations which demand evidence from the youth who is entering upon the study of medicine that he has availed himself of these means. It is against the *exclusive* system that we contend.

It does not seem to have occurred to those who advocate the superior value of ancient to modern knowledge, as a means of intellectual discipline, that the greatest minds of former ages—Aristotle and Plato, Cicero and Pliny, *et hoc genus omne*—were developed upon a literature which *to them* was *modern.* And it has been in times when the wisdom of the ancients was too exclusively studied, that the greatest intellectual darkness has prevailed. It may be thought that we are disposed to dwell too long upon this subject; but the system to which we refer is so strongly upheld by the props of prejudice and established usage, that we fear it will resist vigorous and well-directed efforts to overthrow it, in spite of the rottenness of its foundations. Unless a decided taste is manifested for classical study, or, on the other hand, there is an obvious deficiency of those mental qualifications which this study is most adapted to induce, we do not regard it as advisable to persevere in it beyond the age of sixteen, seventeen, or at most eighteen years in the average of young men. Two subsequent years may be advantageously devoted to the acquirement of scientific and general information, and the cultivation of literary taste, by attention to the standard literature of our own country, so much neglected in the training of English youth. This, perhaps, will be the most advantageous period for exercises in composition, so valuable (under a judicious instructor) in the formation of habits of thought, as well as in the acquirement of power and facility in expression. We know many who look back upon such exercises as the most permanently useful portion of their early training; and a strong testimony of the same purport is borne in the Autobiography of Dr. Franklin, who ingeniously supplied the want of a competent instructor by a more careful study of his author. A portion of the same time may be advantageously devoted to the study of foreign languages and literature. Of these, French and German are of course the most desirable; but the Latin scholar will have so little difficulty in acquiring a tolerable knowledge of Italian and Spanish, that they should not be regarded as by any means out of his reach. Those, however, who have not an aptitude for the study of languages should not attempt too much.

Now, we think, it cannot be doubted that a young man thus prepared, having the advantage of a large amount of available knowledge as well as of high general intellectual development, will commence the study of medicine at the age of eighteen, nineteen, or even twenty, with the probability of much greater ultimate success (if his mind be decidedly bent on the study) than he who is bound an apprentice at the age of fifteen or sixteen, and has passed three or four years in the acquirement of desultory and ill-digested knowledge of some of its least important details. But it will be necessary that he should *then* apply himself vigorously and almost undividedly to the object. We believe that he will *then* be likely to do so from inclination as well as from necessity. If he have acquired that earnest desire of truth, which we have regarded his previous course of study as adapted to excite and cherish, he will not stop short with a superficial knowledge of the phenomena which his habit of active observation will bring under his notice; he will desire to consider these phenomena in all their bearings; and the time not occupied in systematic study will be employed in those spontaneous efforts, which every one who looks back upon the history of his education must feel to have been, if rightly directed, the most valuable part of his training. And this leads us to speak of a *principle* applicable to all education, but more especially to the study of medicine. We deem it of great importance that the growing mind should be caused to *educate itself* as much as possible. We do not go so far as to say that a child should never be *compelled* to learn—a position maintained by some of the new lights of the present day; for we consider the discipline of early instruction as alike necessary in itself, and valuable in preparing for the rough discipline of the world, which must sooner or later be exercised. But in proportion as the reasoning powers expand, and the value of knowledge for its own sake is perceived, will compulsion become less necessary; and the necessity of it will be superseded by voluntary effort. The earlier the pupil can be induced to make this effort, provided that he is still content to follow the guidings of experience and matured judgment, the more permanent will be the benefit. Now it is not among the least of the advantages of such a system of preliminary education as we have recommended, that he who enters upon the study of medicine with a decided preference for it will be disposed and prepared to take his education chiefly upon himself. *Rerum cognoscere causas* will be his motto; and he will find no want of opportunity for carrying the principle into extended operation. That a systematic *curriculum* should be pursued we do not hesitate to affirm; but we feel satisfied that the chief advantage of this is rather to point out the path to the student than to conduct him along it. If left entirely to himself there will of necessity be a certain degree of desultoriness in his knowledge. On some particular subjects which have attracted his especial notice, he will have obtained a valuable, perhaps even a remarkable extent and accuracy of information; on others he will be as deficient. We deem attendance on lectures which are not demonstrative chiefly valuable to the student, as *connecting* the knowledge he may have acquired from other sources, and indicating to himself the points on which he is most deficient. But upon this topic we shall hereafter dwell, when we speak of the individual branches of medical science, and the mode in which a knowledge of them may be best acquired.

By the term medical science we mean that department of knowledge which includes the principles and facts that are subvervient to the *healing art*, and to the prevention as well as the cure of disease. We recognize no distinction at the outset between medicine and surgery. But it must be remembered that, when the practical applications of science are made the ground of its subdivision, a very different view will be taken of the relations of its various branches from that in which the abstract is the true one. Hence it is that, from the importance of a certain amount of chemical knowledge to the practitioner, the study of chemistry has been regarded as so far an essential in medical education, that it has been put on a level with topics of far higher importance. Still more remarkable has been the estimation in which botany has been held. It will be scarcely believed in future ages that full courses on these two subjects are required by the examining boards of the English metropolis; at the same time that it is left to the option of the student to acquire a knowledge of the natural functions of the human body, with whose morbid conditions he is expected to be familiar. In the view which we shall now give of the objects and relations of the various branches of medical science, we shall endeavour to combine the *abstract* with the *practical;* and we feel convinced that whatever system is based on the truest principles will be found also to work the best. What may be the value of the principles we shall endeavour to unfold, it will be for our readers to judge.

We pointed out in our former article that medicine is but a branch of the vast science of biology—that which treats of the vital actions, normal and abnormal, of all classes of living beings. It is evident, therefore, if our first principle—of proceeding from the *general* to the *special*—be correct, that some acquaintance with the latter will be the best preparation for the detailed study of the former; such an acquaintance we suppose to have been gained during the preliminary training, and the study of medicine commences with the more detailed examination of the structure and actions of the human organism. Taken in its most enlarged sense, the term *physiology* may be applied to the whole science of *normal vital action*, and the term *pathology* to that of *abnormal action.* All vital action is dependent upon two classes of conditions: the organic mechanism, which manifests the changes in question, and the external agents by whose operation they are excited; just as the movements of a steam-engine are caused by the action of heat, cold, &c. upon an apparatus fitted to respond to their influence. The structure of the human body and the properties of its various parts, therefore, afford the first objects of scientific enquiry to the student; and it is most fit on every account that anatomy should be the first department of medical science to which his attention should be enforced.

Anatomy may be taught in several different modes, each of which has its peculiar advantages. As subservient to physiology, the structure and arrangement of the organs composing the nutritive system will naturally be first investigated; and then that of the nervous centres and the other parts of the system of animal life. This constitutes physiological or functional anatomy; and it appears to us important that it should be early studied with close attention. But it will not be enough to study this only. The physiologist attends chiefly to the different forms of structure as existing separately; he studies the nervous system, the vascular system, &c. not according to their local distribution, but according to the organs they supply. The particular disposition of the viscera in the abdominal and thoracic cavities is to him a matter of little importance, provided that he knows their functional connexions with each other. The stomach might lie on the right side and the liver on the left, without their respective actions being appreciably changed. But by the *practitioner*, whether of physic or of surgery, much more than this is required. He must study the different organs and systems in their relation with each other. He must know not only the distribution but the course of nerves and arteries; not only the origin and insertion of muscles, by which their respective uses are determined, but the position of their bodies in regard to one another and to the nerves and vessels which pass amongst them; not only the functional but the local relations of the organs contained in the cavities of the trunk. The necessity for this knowledge is so obvious that we need not dwell upon it: as anatomy has been usually taught in our own country this has been made almost the sole object; and herein is the great error of the prevalent system. Such knowledge is indispensable to the practitioner; but its use lies only in enabling him to make a right application of principles. In order to acquire the principles, his studies must be more extended. We will take, for example, the treatment of aneurism. How few students are there who do not regard it as their chief duty to learn the minutiæ of the various operations that may be required, and to impress on their minds the details of the anatomy of the region concerned, neglecting or treating with superficial attention the nature of the disease itself, the peculiar properties of the tissues it involves, and the principles upon which its treatment should be conducted? And, in consequence, what eagerness is manifested to get hold of "a case," on which the young operator may try his *hand* instead of his *head!* Every one knows how many more operations are performed by young surgeons than by those advanced in years and experience, who have the same field of practice. We are much disposed to attribute this in part to the stress laid upon *regional* distribution in anatomical teaching, when compared with the importance attached to those details of structure and properties, upon which all *principles* of treatment must be based. We would not be understood as undervaluing the former, or as overlooking their absolute necessity to the well-educated surgeon; all we contend for is that *principles* should be taught first, and the mode of applying them subsequently demonstrated. At present the student learns the operation for aneurism before he knows what an aneurism is; and, in consequence, the idea of an operation is constantly associated in his mind with that of the disease, inducing him to attach much less than their due weight to those considerations which ought to guide him in deciding upon the treatment of each particular case. And it is not until years and experience have matured his judgment that these are fully comprehended.

We have selected aneurism as a convenient subject for illustration; many others might have been adverted to. But, it will be said, it is easier to find fault than to amend; and any system of teaching must be attended with so many difficulties and imperfections that it is scarcely worth while to change one which on the whole works so well. The experience of the last few years, however, furnishes indications which may be advantageously made the basis of more extensive improvements. And, as anatomy must ever occupy, on account of its demonstrative character, a prominent place in all systems of scholastic instruction in medical science, we shall take leave here to present our ideas on the best mode of teaching it. We shall find in the outset a necessity for multiplying the present number of classes; but we shall hereafter point out the facility and propriety of, at least, an equivalent reduction in other subjects.

We propose, then, as an important addition to the present scheme of tuition, a general introductory course of anatomy, which should serve as a preliminary to all subsequent study of the details of medical science. The course should embrace such an outline view of the structure of the human organism as would be engrafted with propriety on the previously-acquired knowledge of the structure of animals in general. The elementary structure and properties, and the functional connexions of the several organs would be dwelt on rather than their local disposition; and these would be compared with the analogous phenomena presented by the inferior organisms. Upon this foundation should be erected a systematic combination of the general principles of physiology and pathology, including an account of the chief alterations to which the several organs are subject, in respect to their elementary structure and properties, and their functional connexions; and the influence of external agents upon them, either in exciting or modifying their healthy action, or in affecting their diseased conditions. In contemplating the utility of such a course, and the possibility of including in it all that we have indicated, the previous preparedness of the student, both in regard to the amount of knowledge acquired and the aptitude to receive and apply principles, which we think might be generally reckoned upon, must not be left out of view. And, even if it do not effect all we anticipate, it will at least serve to communicate to the student a much clearer idea of the nature and objects of his science than he at present gains. We are by no means sure, however, that *lectures* of this kind are essential. We cannot doubt that they are preferable; since we regard it as of great importance that the student should be familiarized as early as possible with the actual objects with which his subsequent minute acquaintance is required; such as the natural aspect, size, form, &c. of the several organs of the body, and especially of the viscera. But by the country student, who is placed out of the reach of lectures, such knowledge may be gained from a book adapted to communicate it, provided that he avails himself of the opportunities of post-mortem inspection that may fall in his way, to gain that which *sense* only can convey; more especially if he have the advantage of a judicious instructor.

Whilst attending such an introductory course, the student may advantageously prepare himself at home for more detailed anatomical study, by familiarizing himself with the osseous system; a thorough acquaintance with which will lighten many of his subsequent difficulties. We should also recommend that he familiarize himself with the general phenomena of disease, and study the aspect, position, complexion, pulse, temperature, &c. of the patients he may have the opportunity of thus noticing. If he have not an ear well adapted for the discrimination of sound it would be advisable that he should devote himself much to auscultation at this period, in order to familiarize himself with its delicate phenomena; but he should commence this upon the healthy subject, and study the natural sounds of the heart and lungs in as many varieties of age, sex, conformation, &c. as he can command, before proceeding to examine, with any other purpose than that of contrast, their abnormal phenomena. We lay stress upon this, because in auscultation, as in every other department of study, it is easier to prevent the formation of early erroneous impressions than to eradicate them when inrooted; and nothing is so common as an exclusive dogmatism in regard to its many contested questions among those who have not gone through the preliminary training we have urged.

We are ourselves favorable to the nearly equal division of the *annus medicus* into two sessions of about five months each; and such a course as we have been suggesting might very properly occupy the first summer. The slight amount of dissection necessary for it may be easily performed at this time; since it would not be necessary that the subjects should be long kept. The time not occupied in the pursuits we have marked out might be devoted to the completion of previous studies—scientific and literary; and we had much rather that it should be so employed than that the objects of attention should be too abruptly and completely changed. That they usually are so appears to us a great evil in the present system. By the following winter the student will be prepared for a more detailed course of anatomy as well as for other pursuits. In assigning an order to these we shall be guided by their more or less direct bearing upon the grand object of the whole educational training—preparation for practice. In order to make our views more readily comprehensible, we shall revert to the distinctions we formerly drew (Vol. IX. p. 130) between medicine and surgery considered as *arts* and as *sciences*. The science embodies the principles; the art consists in the application of those principles. The more perfect the science the simpler will be the rules by which the artist may work; and where its principles are of the highest comprehensiveness, applicable without difficulty to every case, the practice of them requires more operative skill than sagacious judgment. But the low state of medicine as a science places the practice of it as an art upon a very different footing. No principles of any high degree of generality can be said to have been established; and no rules of practice, therefore, exist, which can be applied without exception or qualification to individual cases. And the complexity of the conditions which affect the phenomena of disease renders that knowledge of its actual state, which must be acquired before any but empirical means can be employed for its cure, one of the most difficult among the objects to which the attention of the practitioner is directed. That kind of practice which has been described as a *rational empiricism*,—admitting principles so far as their value is sanctioned by experience, but regarding the peculiarities of each individual case as the sole guide to be safely trusted in the application of them,—must long, therefore, be the one most adopted by the intelligent members of our profession. And the preparation for it requires not only a knowledge of principles but the study of that mass of important facts which experience has accumulated, as furnishing our best guide in all the details of treatment.

Now, as in considering any individual case, we should first revert to the general principles which bear upon it, and then turn our attention to the special circumstances which modify their influence, so does it seem natural and right to act in acquiring the information which is to be thus employed. For how can the true influence of these modifying circumstances be understood, when the principles to which they are in relation are not known? Considering *practice* itself but as continuance of education, the propriety of so arranging the course of study that the theoretical should gradually merge into the practical, and this again into the actual employment of the knowledge previously acquired, becomes still more evident. Were time unlimited, this plan might perhaps be rigorously pursued; but as it is an important object that as much information as possible should be acquired in the briefest period, it will be necessary in some degree to depart from it. Still it may be taken as the chief guide. The first winter session should afford to the student the means of instruction in physiological anatomy and physiology, which may be included in one course of lectures, nearly corresponding to that now given in University and King's Colleges, London, and also in other schools. In this course should be considered more fully than in the former one those details of structure which obviously furnish the conditions of vital action—the influence of external agents in calling their properties into operation—the general laws governing the phenomena thus induced—and a history of the principal phenomena themselves. For this course, also, the student would have been usefully prepared by his preliminary education; and it would itself serve as the foundation for subsequent instruction of a more practical character. Had we been writing five years ago upon this subject, we should have thought it desirable to insert here a formal disquisition upon the importance of physiological knowledge, and to occupy some time in demonstrating that no medical education can be regarded as complete which does not include a regular survey of this department of science. But the great and increasing demand for physiological works, the success which has attended the change in the character of the anatomical lectures effected in many of the London schools, and the interest which has been shown in our own articles on the subject, lead us to believe that this may now be dispensed with, and that we may start at once from the Q. E. D.

The question may be not improperly raised, whether physiology may not be learned as well from books as from lectures; and we are not disposed to think that oral instruction is by any means necessary. In so far, however, as it is taught demonstratively, lectures are certainly preferable. The instructor is, or ought to be, provided with many means of illustration which the student cannot command; and although plates may to a certain extent supply the want of actual preparations and microscopic dissections, it is always advantageous that *the real thing* should be submitted to personal examination. There is another point in favour of attendance on physiological lectures in preference to an exclusive study of books, and this, which is applicable to the general system of academical instruction, is especially important when considered in reference to a science so rapidly advancing. We refer to the superior opportunity which the annual repetition of a course of lectures affords for communicating to the learner a proper idea of the *actual state* of science at the time. The rapidity of the sale of a book must regulate the degree in which this end can be attained by the introduction of improvements into its text; and too often it happens that a writer thinks more of the mode in which he can engraft his additions, or make his alterations, with the least trouble, than of that which will make his work a condensed reflection of the state of knowledge of the subject on which he writes. The lecturer has not the same difficulty; and if he be a conscientious instructor, will make a point of enabling his pupils to keep nearly on a level with the progressive elevation of his science. That he may do this, however, he must devote a large proportion of his time to the study of it. For it is not enough that he should make himself well acquainted with all that is known at any one time; he will find that it will be constantly necessary to remodel entirely various divisions of his scheme, as new and more exact information supersedes that previously in his possession. It is not so much in the discovery of new phenomena that we are to look for the advancement of physiology as in that more exact knowledge of the nature and conditions of those already under our notice, which may lead us to a more certain acquaintance with their causes and laws. The man who is immersed in practice, and who feels it necessary to keep up with the advance of knowledge of a solely practical character, cannot be expected to be a good physiological teacher; nor, on the other hand, do we look for consummate excellence in one who should devote himself too exclusively to physiology. The close connexion which is manifested between the healthy and morbid phenomena of living beings not only requires that a good pathologist should have a firm basis in the knowledge of normal life, but that the devoted physiologist should maintain his familiarity with the phenomena of disease. Each class is equally necessary to be taken into account in the study of the other; and, accordingly, the education of the medical man should be the same whether he devotes his subsequent life to the pursuit of the science or the practice of the art.

The physiological teacher ought to be well provided with the means of illustration, by delineations, where the objects themselves cannot be exhibited; by recent specimens and preparations to as great an extent as possible; and by the exhibition of microscopic dissections and preparations of minute structure, and of the phenomena of vital action, where these can be readily shown. We shall not discuss at any length the mode in which the course should be distributed among its several subjects. It ought to include a comprehensive survey of the general laws of vital action; a comparative view of the different groups of phenomena or *functions*, as exhibited in the principal classes of living beings; and a detailed examination of them as they occur in man. Particular attention ought to be paid to the classification of these phenomena in regard to their abstract character—whether simply physical, entirely vital, or of a mixed nature. Experimental illustrations may often be advantageously introduced, for the purpose of showing the bearing of these upon each other, and the operation of physical causes in the production of phenomena which at first sight appear due to vitality alone. But we enter our earnest and decided protest against the practice of repeating before a class, for the sake of illustration only, experiments which involve a painful sacrifice of life or happiness in sentient beings, however low in the scale. We are not so strait-laced as to object to such experiments in the abstract, when made for the purpose of establishing important truths, but we are confident that much animal suffering may be saved by *first* ascertaining all that *observation* alone can reveal with reference to the subject, and putting questions to nature only when no other means of information are accessible. That this is often necessary we freely admit; but when once the desired results have been obtained we cannot but consider the needless repetition of the experiments as an unjustifiable cruelty. And in no instance does it seem to us that the class-room can be the proper scene of their performance. That degree of freedom from disturbing causes which is essential to the fidelity of almost every result can only be obtained amongst a small number of bystanders; and we cannot but regard that familiarity with animal suffering which is induced by the frequent contemplation of them as of dangerous influence to the moral sensibility of the student, blunted as it almost necessarily must be by the scenes of the dissecting-room and operating theatre.

In the physiological course should be included all the generalizations and the most important facts of animal chemistry. For these it appears to us that the student will have been sufficiently prepared by the preliminary course. We cannot but regard the amount of attention to chemical science required by all our medical boards as a great error. The extreme remoteness of its connexion with practice is quite sufficient, in our minds, to destroy its claim to be considered a branch of medical education. That its general principles and most important facts may be advantageously made a branch of general education we have already shown, and we do not see what more than these is required from the medical practitioner, except in a few particular departments, to which his special attention will be directed. In the course of physiology, for example, he will study the chemistry of the healthy animal solids and fluids; in that of pathology, the principal alterations which these undergo in disease. In studying materia medica, he will be sufficiently instructed in pharmaceutic chemistry, and his attention will be directed to the operation of general principles in those rules of prescribing by which the combination of *incompatibles* is forbidden. The teacher of forensic medicine will explain to him the application of chemical tests in the detection of poisons, blood-stains, seminal fluid, &c. And in the courses on the practice of medicine and surgery, the student will learn the very few facts relating to the application of chemical principles in the treatment of disease, which the present state of knowledge allows to be positively stated.

Now in the place of further argument on the abstract want of connexion between chemistry and medical practice, we shall satisfy ourselves, and we think convince our readers, by appealing to experience. Who but those who study chemistry as their regular pursuit find themselves now able to keep up with its rapid advance? What medical practitioner who attended a course or two of chemistry a dozen years ago knows anything of the *present* state of the science? Who that has devoted himself to the toilsome labours of his profession for a few years remembers more than those general principles which *our* plan would equally communicate and as permanently impress? A surgeon of *twenty* years' standing has little more than a vague notion of the atomic theory; one of *ten* hears of the sulpho-salts,—the double chlorides, iodides, and fluorides,—the compounds in which water acts as a base or an acid,—of paraffine and eupione, of mercaptan and camphene—with a feeling of indifference if chemistry were never a favorite object with him, and of despair if it were; and one of *five* abandons all hope of keeping pace with Faraday's discoveries, and hears of Dumas's law of substitutions, which is to revolutionize the whole science of chemistry, with much the same feelings that would be excited by an account of a change of government in Columbia. Now what does all this prove but that it is absurd to require from the medical student more than an acquaintance with those general principles which constitute the whole of the real knowledge which he possesses when he has been for ten years in practice? If he can do as well without more extended and detailed knowledge, is it not much better that his time should be bestowed upon other subjects more immediately connected with his subsequent occupations? All will agree, we think, that he *cannot* give *too much* time to practical study; and as the whole period is but limited, it seems better to exclude from it all that is not essential.

It is but natural that every teacher should have a strong conviction of the importance of the subject to which he devotes himself, and we know many who think that the education of no medical man can be regarded as complete who has not gone through a full course of experimental study and quantitative analysis. But let the importance of chemical skill and knowledge to the mere practitioner be compared with that of other branches of science, and it will be at once seen that to bestow the same proportional attention to *them* would require that the course of preparatory study should extend over the whole lifetime. It is most true that circumstances *may* arise in the experience of every one to render such knowledge available or even desirable, but what kind of knowledge is there *not* of which the same might not be said? We remember once to have heard it brought forward in a youthful debating society as a weighty argument in favour of field-sports that the skill of a "good shot" had sometimes been exerted in preserving not only his own life but that of his companions, when cast away by shipwreck; and that consequently it was incumbent on every one to learn how to bring down a bird on the wing, for the chance of his being, at some part of his life, thrown ashore on a desert island. Every one must perceive that preparation for daily, hourly duties is "the one thing needful" in medical education; and that the less frequent cases which require knowledge and skill of a peculiar kind will be much better referred to those specially qualified for them than imperfectly disposed of by one whose opportunities of information on those points are few and far between. We do not think that chemical science will suffer from the plan we have proposed; for by including it in general education a much larger number of youths will be led to pay that moderate degree of attention to it which is more likely to lead to the formation of a decided taste for it as a regular object of pursuit, than the compulsory study of its technical details. And those who have seen how frequently the medical student is seduced into an undue devotement of time and mental energy to chemistry, during the short two or three years of his attendance on lectures, withdrawing them from that which is of so much more practical importance, will be satisfied, we feel confident, of the propriety of the change we have advocated.

Similar remarks will apply to botany, a science whose present position in the medical curriculum is entirely due to the persistence of ancient usage and prejudice. The healing art, in its early epoch, consisted of little else than "skill in herbs," and with the botany of that age it was therefore almost synonymous. As the materia medica became more extensive, and the practice of the healing art at first more limited to a few, and subsequently more divided into separate departments, the herb-gatherer has been separated from the herb-user; and the substitution of regular pharmaceutical preparations for the extemporaneous compositions formerly employed, has still further increased that distance. At the same time the character of the science of botany has undergone great modification. For a knowledge of the properties of plants, to which that of their forms was only subservient, has been substituted a minute investigation into their internal structure, their physiological actions, and the relations of their several forms to each other in a natural classification. Hence, from being originally almost coincident, the objects of medicine and botany have become widely separated; and we cannot see any good reason for maintaining the present connexion between them. We have supposed that in his preliminary education the student has become acquainted with the general principles of botanical science. He has been led to examine the structure of the principal tribes of plants, and to perceive their relations with each other; he has also gained a general acquaintance with their physiological actions, by which he has been prepared for the more detailed illustrations which they furnish in certain departments of human physiology. Now this knowledge is all that he will be likely to retain after a few years, supposing him to have been taught more, unless he makes botany an object of especial attention; and on this account we say, as in regard to chemistry, that it would be much better that his attention be not occupied with its details, at a time when there is so much other and more important employment for it. In the course of materia medica a botanical account of the chief officinal plants may be easily included; and as these belong to a small number of natural families, the student will have the opportunity of making these, which are alone of real importance to him, the subject of more detailed examination. In studying forensic medicine too, it will be desirable that he should familiarize himself with the appearance of the chief poisonous plants of his own country; but further than this we cannot see that he *need* go. As the supply of medicines is now conducted, the practitioner rarely sees the original herbs: he is supplied with the active principles, the alkalis, the extracts, the tinctures, by his druggist, and for their character he is entirely dependent upon him. Of what consequence is it to the practitioner to distinguish the three varieties or species of cinchona, provided that his sulphate of quinine is good? or to pronounce upon the merits of a sample of senna when not an ounce of it enters his house save for domestic use? As the original race of apothecaries dies out, such matters are left to the wholesale druggist and professed pharmaceutist; and with these they had better remain, provided that sufficient qualifications be required for them. We do not deny that a knowledge of botany may occasionally be useful to a medical man, but these occasions are rare, and are principally confined to those living amongst an imperfectly studied vegetation, where new plants offer themselves to the enquirer for therapeutic agents, towards the properties of which they may be guided in some degree by the knowledge of the natural order to which they belong. But to those who will thus have a reason for seeking an increased acquaintance with botanical science, it will be easy to extend their knowledge of it, if a good foundation have been originally laid. And it is scarcely worth while, for the chance of such benefit from it, for the busy medical student to turn his thoughts to it, otherwise than as a healthful and pleasant recreation. We will only ask our older friends whether they do not see the justice of our remarks, and whether, prejudice apart, they do not agree with us that this time-honoured connexion should now be severed.

Putting aside then these two objects, which usually occupy a large part of the student's attention during his first year, we proceed to enquire what may at that period be most advantageously substituted for them. It appears to us that during the time of his attendance on physiological lectures, he may advantageously employ himself in the private study of hygiène. This department of knowledge is scarcely to be regarded as a distinct science, as it is but an application of physiological principles to the various conditions in which man is liable to be placed. The *art* of preserving the body in health can only be perfected by an entire acquaintance with the conditions of its vital action, with the injurious as well as natural operation of external agents, and with the best means of avoiding or counteracting all deleterious influences which arise either from these or from its own peculiar susceptibilities. In proportion as the scientific principles of physiology are understood will the art of hygiène be perfected; and in proportion as that art is carried into practice will the liability to disease diminish. When we see how constantly the most obvious truths, the most important rules, are disregarded, the only wonder is that health is not more frequently and irreparably impaired. Even among those who are looked upon as well-educated medical men and judicious practitioners there may be too often observed not only a total neglect of hygiènic indications, but even a perverse opposition to them. We can only account for this by remembering how exclusively their attention has been directed, during the period of their education, to the phenomena of disease, and how little occasion they have felt to revert to its simple precepts when heroic remedies are so abundant.

Thinking as we do that it is much better for the student to divide his attention between lectures and private study, than to devote himself to either exclusively, we are of opinion that hygiène constitutes a subject particularly adapted to the latter, whilst physiology is being taught by the former. There is at present no work in our language that can supply what is required by the very judicious regulations of the London University; nor are we acquainted with any which nearly approaches our idea of what such a work should be. Of the many books, excellent in their several ways, with which our press has teemed during the last few years, all, we believe, are addressed to the public rather than designed for the instruction of the medical man. Such a treatise as we should wish to see could only be executed by one who should combine with profound physiological knowledge a fund of practical information derived from long experience and from acute and sagacious observation under a variety of circumstances. Dr. Combe could write such a book.

Whilst the student is devoting his chief attention to physiological anatomy and physiology, which are to furnish him with the principles that should guide him in the conservation of health, and in the investigation of disease, he should not withdraw himself from the pursuit of knowledge of a more directly practical character. He should employ himself much in the observation of morbid phenomena, contrasting these with the healthy condition, and accustoming himself to a regular system of examination, which is so valuable in diagnosis, and furnishes so many important and frequently unexpected data for the establishment of principles. It is very desirable that he should not at this period acquire any more than *very general* notions on the subject of their *treatment.* We have already adverted more than once to the strength of first impressions; and in no case is this more remarkably displayed than in the influence of the practice of the first instructor to whom the student looks up as an authority over his whole subsequent career. On this account we much desire that the student should make himself well acquainted with the phenomena of disease, and gain a general idea of the influence of various therapeutic agents, before he is led to put faith in the efficacy of any one system of treatment; and that his peculiar attention to *practice* should not be given until a later period, when he will have learned from his more extended reading and from lectures how much he will have to trust to his own judgment in the selection of means of cure, and how little he must be guided by authority. The most advantageous practical exercise which the student can perform at this period is the observation and recording of well-marked cases of disease; and if at the same time that he observes he compare his own experience of individual cases with the general description given in standard works, he will be led to attend to the points of agreement and difference and to trace the common bond which unites many varieties together, whilst he endeavours to find out some reason for the discrepancy. In this way, if his opportunities for observation are tolerably extensive and his cases well selected, he will have acquired in a few months a valuable amount of information on the phenomena of disease as well as trained himself in habits of accurate and sagacious observation. There appears to us an important advantage in the plan we have thus suggested over the mode in which the study of medical practice is commonly pursued,—that by chiefly devoting himself at this time to the investigation of the phenomena of disease, in which he may be safely guided by the records in his possession, he is entirely independent of the practical ignorance or dullness of the instructor under whom he may happen to be placed, and will not learn anything which he will have to unlearn at a later period. Disease is much the same everywhere; practice is widely different. Many a bad habit is acquired in the early part of pupilage which it takes years of subsequent discipline to eradicate; many a prejudice imbibed which exerts an almost imperceptible, unacknowledged influence to the end of life.

Another portion of the first winter session should be given to the study of Descriptive Anatomy. Considering it simply in the abstract we should not place it here, since it bears more upon the application of principles to practice than upon the principles themselves; and the establishment of the latter upon the basis of physiological anatomy should in strictness precede the former. But every one knows the difficulty of retaining in his mind the unconnected details which the knowledge of descriptive anatomy involves, and the necessity of a repeated study of them for the durability of the impression. We do not see that in lectures on physiology and descriptive anatomy, the former being connected with the private study of hygiène, and the latter with a moderate amount of dissection, we have provided more than the diligent student may readily accomplish, even whilst giving a regular amount of time to the observation of disease. Much will depend, as we have several times remarked, upon the previous habits, and where the mind has been trained to bestow steady attention upon the object before it to receive with intelligence and to retain with philosophic grasp, it is amazing how much may be acquired in a short time, provided that a methodical and well-arranged plan be judiciously pursued. We would not have the student devote himself, however, with too great earnestness to the acquirement of anatomical details at this early period. The structure and local relations of the viscera and the distribution of the nervous and vascular systems among them should occupy his chief attention, since the knowledge of these will be of great importance to him in the investigation of disease. If he be prepared by a thorough acquaintance with osteology, the general arrangement of the muscles, and of the blood-vessels and nerves which lie among them, will be comprehended and retained without much difficulty, and this we think is as much as he should first attempt. In studying the attachments of the muscles, their physiological action ought to receive especial notice, since a sound knowledge of this is of such frequent and important advantage in the treatment of fractures and dislocations of the extremities. Such details are much better fitted for the course of descriptive anatomy than for that of physiology, since they relieve the otherwise dry technicalities of the former, and can be very well spared from the latter.

We doubt not that many of our readers will think us treading on dangerous ground in encouraging the slightest relaxation of the attention now given to the study of anatomy: but we would entreat them to weigh well its value in comparison with that of other branches of medical knowledge, and not to be too hastily led to pronounce us in error by the *dicta* of authorities or by prejudices derived from their own education. Let it be borne in mind that anatomy furnishes, towards the establishment of the *principles* of medicine, only one set of facts, the value of which will depend entirely upon the relations developed between them and others that are brought to bear upon them. To examine ***structure*** without enquiring into *function*, is in this point of view totally valueless: and to a scientific knowledge of function an enquiry into the influence of external agents upon the organism is just as important as that of the structures of the organism itself. Could any one understand the actions of a steam-engine who contented himself with investigating the structure of its several parts or organs, in ignorance of the expansive power communicated to water by heat and the condensation effected by cold? And would any one choose to commit the repair of a complex and delicate watch to a workman unacquainted with its principles of action, however excellent he might be as a mere mechanic? There are those who think it a necessary preparation for the practice of the medical profession that the pupil should get by heart the contents of every cubic inch of flesh in the human body, and who could descant at length on the utility and even indispensableness of such knowledge. But it must not be forgotten that others could talk as plausibly in favour of a complete course of classical study or of chemical manipulation. Let it be subjected to the same practical test which we have before applied to chemistry and botany. To this there can be surely no exception. Let any general practitioner of ten years' standing be asked as to the amount of anatomical knowledge which he retains, and of which he finds the use, (and he will soon forget all the rest,) and we venture to say that our objector, supposing him to be an anatomical teacher, will be rather startled at its small amount. We are far from deprecating any extent of anatomical knowledge which the student has time and inclination to obtain, but we think that common sense indicates the propriety of not *cramming* him with more than experience shows to be permanently valuable, especially during the first year of his pupilage, when we would have him chiefly or solely impressed with those truths which are to be of most importance to him in after life. That there *may be* circumstances in the life of every practitioner which may render the minutest anatomical knowledge desirable, and that irreparable injury *may* possibly result from the want of it, all must admit; but are we then to regard such knowledge as imperative upon *every* student? We should, upon the same principle, require from him a *perfect* knowledge of medical, surgical, and obstetric practice, which it is quite Utopian to expect. The "non omnia possumus omnes" cannot be too steadily kept in view in forming plans of medical education; and when the great extent of the domain to be surveyed is remembered, and its deep intricacies are borne in mind, the importance of as much simplification as possible in the objects and amount of the knowledge to be gained becomes sufficiently apparent. Now let any one contrast the *possible* mischief that may result from ignorance of some difficult points of minute anatomy with the *certain* and *constant* evils resulting from the transgression of hygiènic rules permitted and even enjoined by medical men to their patients, and there cannot be a doubt we think of the advantage of attending to the latter, even at some loss of the former. The anatomy of the chief surgical regions cannot be too thoroughly known; and though we deem its proper place, as we have already hinted (p. 181,) to be in a later part of the curriculum, where it stands part of the surgical course, it ought not to be neglected at an earlier period, in order that it may be strongly impressed on the mind. But as it is very seldom that the mere general practitioner *need* perform those operations for aneurism, stone, &c., which require the greatest anatomical knowledge, it appears to us that the preparation for them may be mostly left to those who decide upon devoting their chief attention to surgery, and who will go through a higher course of education for this express purpose.

The next branch of medical science which presents itself to our notice is Pathology—the science of *morbid action ;* and to this and its allied topics the student may advantageously devote himself for a considerable period. In the sense in which we employ it, the term pathology may be regarded as of parallel import to physiology, including the consideration of all the abnormal states of the organism, whether congenital, accidental, or acquired ; of the external agents which may operate injuriously upon it ; of the irregularities of action thus resulting ; and of the various influences by which these may be checked and the normal state restored. We thus include the departments known as morbid anatomy, ætiology, and general therapeutics, in addition to the science of disease or nosology and symptomatology, as commonly understood. To the study of these, concurrently with that of actual phenomena, we would have the pupil give his almost undivided attention during the second summer session ; and under the guidance of a judicious instructor he may pursue this as well from books as by attendance on lectures, when at least such books as we should wish to see shall be in existence. In studying pathology, we should not perhaps find it convenient to pursue the order which abstractedly is most scientific. The diseases which can be traced to original and evident peculiarities in the structure of the body are but few and not of primary importance. It would be better to commence with an exposition of what is known of the general influence of local changes on the constitutional state, and of the converse influence of constitutional states or diatheses in producing local diseases, the express seat of which is determined by accidental circumstances. Let it be remembered that we use these terms only to express conditions which we know by their results, and of the hidden nature of which we may gradually learn something by a judicious mode of enquiry. Every one knows that there are such predispositions, and we can see no objection to the use of the term *diathesis,* if it be borne in mind that it really tells us nothing of the cause of disease but expresses only that which a little circumlocution would convey in common language. The influence of the natural external agents on peculiar states of the organism and that of peculiar morbific agents on the organism might then be discussed : these two subjects together constitute the science of ætiology, which obviously holds a corresponding place in pathology with the laws of the operation of the vital stimuli in physiology. From this we should proceed to study the principal classes of diseased actions in general—their phenomena, results, and the conditions on which they depend. Here it will be seen how small a part is morbid anatomy of the real science of pathology, and how much attention needs to be given to the phenomena of disease as manifesting themselves during life, before we can become scientifically acquainted with it. It is necessary to bear in mind the distinction between an elementary or simple morbid action considered in itself—such as inflammation, and that train of sympathetic phenomena resulting from it which constitute what is commonly regarded as the disease, the symptoms being mostly due to the latter. In order properly to appreciate both they must be studied separately; and the groups of phenomena which manifest themselves under the joint influence of both may be afterwards detailed. The nature of the action itself having been discussed, its results should be enquired into: and here it is that morbid anatomy becomes most useful. But that the information which it imparts is at best very imperfect is evident from this, that violent diseased action may have existed during life without leaving any traces perceptible to attentive scrutiny, if its duration has not been sufficient, and that, on the other hand, appearances may often be discovered after death which have no relation with any diseased action occurring during life, and which nothing but a very sound discrimination can distinguish from those which are really indicative of it. Moreover there can be no doubt of the existence of many diseased actions which anatomical skill has hitherto failed to detect, and which will probably long continue stumbling-blocks to the pathologist.* Further, it is now generally admitted that the first departure from the healthy state generally, if not always, exists in the fluids of the body, and especially in the blood; and a thorough examination of these, microscopically and chemically, in every case would, if it were possible to make it within a short time after the extinction of life, undoubtedly throw as much light on the nature of disease as the examination of the structural changes in the solids. We fully anticipate that a time will come when the latter will be only regarded as indicative of the existence of minuter alterations of a more general character, of which they will be considered as particular instances, modified by special circumstances; in other words, that the pursuit of the line of investigation which has already been attended with such brilliant success in reducing a large number of apparently different diseases to the same general standard, by the separation of their accidental from their essential phenomena, will be equally or still more fertile in important results, when followed with increased sagacity and extended means of research. We may notice, by way of example, the Protean manifestations of what is now known as the tubercular diathesis; and the relations lately developed between these and inflammatory conditions.

With the general account of the nature and causes of the principal varieties of diseased action may be properly combined a general account of the curative means adapted to each; and this will include a sketch of the operation of different classes of remedial agents upon the system, and of the most advantageous means of employing them. This is usually embodied in the course of materia medica; but we are persuaded that its proper place is here, general therapeutics being strictly a part of general pathology. And it will be useful to the student that his first ideas on the treatment of diseased action should be immediately connected with those of its nature. When more details become necessary they may be advantageously separated. Under the head of special pathology will be considered the combinations or groups of diseased action as they exhibit

* This is especially the case with those in which the nervous sympathies are much involved.

themselves to us; that which is believed to be the original one, or fundamental cause, being first expounded, and then the collateral phenomena displayed. This will lead to the consideration of the symptoms to which they give rise; and the difficulties of diagnosis will be frequently adverted to, although it will be better not to treat of them here in detail. In this department of the science, diseases should be classed according to their abstract nature so far as known; and the subdivisions of these classes will be grounded on the character of the tissue or organ affected. But it will not be desirable to enter here into an exposition of the treatment of each; its causes, however, should be fully discussed.

Such is an outline of the course of instruction in pathology which we should deem at once philosophical in itself and adapted to the wants of the student. If, whilst pursuing it, he devotes himself sedulously to the observation of phenomena at the bed-side of the patient and in the dead-house, he will have acquired an excellent preparation for the study of the practice of medicine, surgery, and midwifery, to which he should devote nearly all his subsequent time. During his second winter he should pursue his anatomical studies; and he should make himself acquainted, either by lectures or reading, with the particular properties and mode of use of the different articles of the materia medica. This knowledge is, we think, best gained from lectures, if the instructor be a man of large experience; but from books if he be not competent to afford original information that can be depended on. And here we may remark, once for all, in regard to lecturers, that, other things being equal, we should consider young men preferable as instructors in medical *science*, and men of mature age and extended experience as teachers on *practical* subjects. The truth of the latter proposition is obvious; some of our readers may differ from us in regard to the former. Our reasons are these:—it is among the young that we usually see most strongly existing that love of knowledge for its own sake, which shall cause them to delight in the advance of knowledge, in spite of the additional trouble which it requires from them to keep pace with it. With those who have long had to endure the cares of this world, and to struggle with difficulties and discouragements in the discharge of their duties, often almost weighed down by their ill-recompensed labours, the *cui bono* very naturally operates strongly; and if no immediate practical benefit seems likely to result from a new discovery, however interesting and important it may be in itself, they do not feel inclined to give themselves much trouble to become acquainted with it. On the other hand, the eagerness of youth takes especial delight in such; and the more unoccupied state of the mind prepares it for the reception of scientific truth once proved to be such, which would have to beat down a strong wall of prejudice raised by long habit before it could enter the understanding of the veteran. We have reason to know that the comments we gave in a former number on Dr. Macartney's opinions on the subject of inflammation were appreciated and adopted by many of our younger brethren, whilst others more advanced could not be driven, by any argument, from what seemed the impregnable position, that no reparation *could* take place without an inflammatory process.

We shall say little upon the instruction on the practical departments of the profession, which should occupy the latter part of the student's academical course, because we have little to suggest in regard to the mode of teaching these subjects which will be new to most of our readers. It appears to us that when pathology has been previously studied as a science, diseases should be expounded by the teachers of practical medicine and surgery, not according to their fundamental nature, but as they present themselves to the observer during life; and thus they will be classified either according to the nature of the symptoms to which they give rise or to the position of the organs they affect. The pupil should be led to observe each as a group of phenomena, by the study of which he may, through a sagacious and skilful investigation, arrive at a knowledge of the latent cause; and having acquired this, he will have to bring experience, either of his own or of others, to determine the best means of cure. We approve of a nosology founded on one or other of the two principles we have alluded to—the fundamental character of the disease, or its external aspect; but we think the mixture of them decidedly objectionable. A practical nosology may be made perfect at once; but a scientific distribution can only be made in a very advanced condition of knowledge; and the two have or ought to have little in common. The more the student is led at this period to observe disease, not in its leading symptoms only, but in those minutiæ on which the success of treatment so often depends, the better prepared he will be to be thrown upon his own resources. We should be disposed to require two or even three years of *practical* study from every candidate for a general licence to practise; and these might be advantageously passed in three different schools. For we deem it a great advantage to a man who has once acquired fixed principles to see as much variety as possible in the mode of carrying them into operation. But the habit of going from one school to another whilst the mind is yet immature, and is unpossessed of the knowledge requisite to discriminate the good from the bad, we deem very prejudicial. If good practitioners were "as plenty as blackberries," we should deem Mr. Jones's plan—that every student, on completing his academical education, should pass two years with a medical man of ten years' standing—a very useful one; but, in the present state of the profession, to introduce it as a legislative enactment would be, it appears to us, completely out of the question. For, with such an education as we have proposed, the junior would of necessity feel his superiority in many respects to his more advanced friend; and their intercourse would thus be rendered very difficult. We should prefer trusting to a well-devised system of clinical instruction and observation, in which that attention should be bestowed on individual cases which, as clinical lectures are now frequently given, is directed to the disease rather than to the patient. According to the plan which we have proposed, the disease would be treated in this manner in the course of practice of medicine; whilst it would have been more *scientifically* studied in that of pathology.

It will have been perceived that all the departments of medical science which we have hitherto glanced at concern man only as an individual. Were a human being placed alone in the world, with a knowledge of the effects of disease and of the means of cure within his reach, he could apply that knowledge to his own case if necessary, as well as to the members of an extensive community if he were situated among them. And as the liability to disease and injury is a part of the inherent constitution of the bodily structure and mental endowment of man, so do we find in all countries and at all epochs individuals who have made the means of his restoration their especial study.

Now, the science of law or jurisprudence is also based upon the constitution of man; but instead of regarding his physical nature, it is principally connected with those moral tendencies which affect his fellow-men. It considers him not in his individual but in his social character, not as an insulated being but as a member of a community. The solitary inhabitant of a desert island owes a moral responsibility to his Maker, but he holds no relation with his fellow-creatures; he exercises no influence over them, he is subject to no restraint from them. Monarch of all he surveys, he freely roams through his domain without the possibility of injuring the person or the property of others, or of receiving injury from them. But suppose such a one transferred to the midst of a civilized community; immediately that he comes into relation with his fellows, a *duty* towards them begins to operate. This prevents him from committing actions detrimental to them; and, in like manner, he enjoys a corresponding security by the influence of *their* moral obligation. Whence this moral obligation primarily springs it is not our present purpose to enquire; but it is evident that the *rights* of man are but its result, and that these rights cannot be said to exist until man is brought into connexion with his fellows.

It is a necessary result of the nature of the social state that every individual must make some sacrifice of his natural rights for the common benefit; but that which he surrenders for the welfare and security of others is repaid to him in the corresponding security in which he is placed by the operation of the laws framed for the benefit of the community. It must be evident to every one that the perfection of a code of laws destined to extend the greatest protection to all, with the least infringement of the rights of any one, must depend upon its adaptation to the physical and mental constitution of man. Here, then, is the original, the fundamental connexion between law and medicine. Whilst the physician studies with minute accuracy every department of the structure and functions of individual man, the legislator seeks to derive from him those particulars which concern man in his relation to his fellows. And as laws for the regulation of conduct can only be securely framed by those who have analyzed the springs of human actions, so those which bear upon the physical condition of man can only be firmly based upon an acquaintance with his corporeal frame. Too many instances of a neglect of these precautions will occur to those who have cast a discerning eye over our statute book; and many laws, fortunately not often called into operation, still disgrace our code, which afford a painfully convincing evidence of the ignorance and empiricism which prevailed in the times when they were framed. One object, therefore, of *legal medicine* (using that term in its most enlarged sense) is to bring the science of medicine to bear upon the enactment of laws, for the preservation of the health and physical well-being of the community. This department of the science, commonly but incorrectly denominated medical police, is now beginning to assume its proper rank in other countries; but our own is far behindhand in this respect; and laws are still enacted in absolute defiance of the plainest hygiènic principles.

Supposing that a complete code of laws had been framed, which should accurately define every species of offence against the person or property of others, and should apply to every case in which the social rights of men are involved, it is manifest that, in the proof that offences against these have been committed, and in the determination of various doubtful questions to which they relate, evidence will be required from those who have made the human frame in health and disease their especial study. It must be observed that, with regard to matters of *fact*, medical testimony is no more valuable than that of an observant spectator; but it is the business of the medical jurist to enquire into many facts which he knows to be important, although trivial in the eyes of others; and to state the inferences to which he is led from them by that previous preparation of mind which alone can enable him to appreciate their true value.

If, then, we regard *legal* or *juridical medicine* as embracing all the relations which subsist between law and medicine, we may consider *medical police* as including those which concern the framing of laws, and *forensic medicine* as comprehending those involved in their execution. This is a good practical division of the subject; and has the advantage of being founded upon the original basis of the whole science.

That in any scheme of medical education which professes to be adapted to the wants of the age, juridical medicine should occupy an important station, will scarcely, we think, be disputed. Medical police and public hygiène are so closely allied that they may be conveniently taught together; but the details with which forensic medicine is concerned are drawn from a much wider range of subjects. Not only physiology but pathology; not only hygiène but the practice of medicine, surgery, and midwifery, with chemistry and botany, ought to be understood by the medical jurist. But it may be said, if forensic medicine only embraces those departments of the various medical sciences which have a relation with the administration of justice, there is no need of its being made a distinct branch of education, as the student becomes acquainted with each group of facts in the usual course of professional acquirements. This, however, is far from being the case; since most of the facts upon which the science of forensic medicine is grounded are of a kind which the mere practitioner would disregard, and which derive their sole importance from their connexion with legal questions. Thus, when an individual receives a wound from some unknown source, it is the object of the surgeon to repair the injury in the safest and speediest manner; while the medical jurist looks for some indication of the weapon with which it was inflicted and the motives which influenced the perpetrator. How successfully the minutest particulars of the case may frequently be determined by a skilful examiner, the records of justice sufficiently attest; whilst, on the other hand, they frequently exhibit instances of ignorance, presumption, contradiction, and neglect, which are a disgrace to the character of the profession.

Abstractedly considered, however, we can scarcely regard forensic medicine as a distinct science. The practice of it is an *art*, based upon principles derived from several distinct sciences; these principles having no relation whatever to each other, but having, from accidental causes, a common direction to the same point. Thus, in the investigation of a case of suspected poisoning, the medical jurist enquires into the symptoms during life as if he were a physician, contrasting them with those produced by natural disease; he examines the appearances after death as if he were a pathological anatomist, and endeavours to draw from them additional evidence; and he analyzes the contents of the stomach, thus obtaining the most decisive of all testimonies as a chemist. In the practice of all departments of medicine there is somewhat of the same combined application of distinct and unconnected principles; but it will generally be perceived that these are brought in as *adjuncts*, and are subservient to some main principle which is by their means to be carried into operation. Thus, the surgeon employs mechanical contrivances in the reduction of dislocations; the physician administers remedies which shall have a purely chemical operation upon the contents of the stomach. But the whole science of forensic medicine is made up of such unconnected materials; and there is no one fact which can be said to afford it an independent foundation. Take, for example, the medico-legal enquiries which are prosecuted in a case of suspected infanticide, and it will be seen that they are mostly connected with the performance of certain physiological changes which constitute a part of the history of the new-born infant. Suppose that we perceive, on looking at the exterior part of the body, that the cuticle is desquamating, or that the separation of the umbilical cord has partly taken place, our minds are at once made up as to the fact of the infant having lived for many hours. What are these signs but the results of important vital changes?—results of no moment to the physiologist and therefore unnoticed by him, but still forming a part of a complete history of normal life. If these signs are absent, the enquirer proceeds to the examination of the respiratory and circulating apparatus; and all the indications for which he seeks are dependent upon the performance or nonperformance of that great physiological change, the most important in the life of the human being, by which the circulation becomes dependent upon the respiration of the individual. Now the physiologist looks only at the effect of this change on the future history of the being. It is of no consequence to *him* whether the ductus arteriosus contracts equally through its whole length or whether it assumes a conical form; whether the lungs inflated by natural inspiration are more disposed to float in water after having been submitted to pressure than those distended by insufflation. But these, although the foundations on which the medical jurist erects his inferences, are really questions appertaining to the physiologist; and the same may be said of a great number of others.

Seeing, then, what extensive acquaintance with the details of other branches of science is required from the medical jurist, it is rather surprising that, in the English schools, this important province should generally be assigned to the youngest and least experienced among the teachers. Its recent introduction as a distinct branch of study will in part account for this strange condition. We must wait a good many years before we see it occupying its due position in this country; and before such medico-legal reports, as those which France and Germany can abundantly furnish, shall be readily obtained from those qualified to kill or cure in Britain. At present there is a great apathy in regard to this subject among a large proportion of medical students. They look upon it as one of little or no practical importance; and one with which it is very likely that they may never have any concern.

Every medical man ought to be so far acquainted with the questions which are involved in the practice of forensic medicine as to be able to commence with discrimination and accuracy the peculiar enquiries which any case that presents itself to him may demand. And as the medical attendant is often among the first persons, capable of giving intelligent testimony brought to the scene of action, he should not omit to notice those collateral circumstances which are frequently so important in the subsequent investigation. But we are of opinion that the further prosecution of the enquiry had better be committed to those more skilled in it. It is impossible that every practitioner should become a skilful medical jurist. It might as well be expected that every one should be an eminent physician, a first-rate surgeon, a profound chemist, a skilful oculist, a learned physiologist. In this department, as in every other, there must be a division of labour. We do not see why a professed medical jurist should not be called in to prosecute a medico-legal enquiry with as much propriety as an eminent physician or surgeon is consulted in a difficult medical or surgical case. This is the custom on the continent, and it is a very common practice in Scotland. The professors of forensic medicine in the Universities are consulted, as a matter of course, in any doubtful case; the contents of stomachs are sent to them for analysis from all parts of the country; and the proceedings on the side of the crown are conducted in accordance with their opinion. This plan, we conceive, would be introduced into England with far more advantage than the medical coroner system. It is as a *witness* that the medical man is important; not as a judge. If the medical coroner get hold of a stupid or blundering medical witness he has no help; the verdict must be given according to evidence; and *he* has no right to contradict the statements which have been given. On the other hand, an intelligent lawyer accustomed to investigations of this kind, and a skilful medical witness, present the combination which is evidently most likely to elicit the truth. In every important provincial school there is now a teacher of forensic medicine; and thus, in almost any part of the country, a skilful witness may be obtained within a moderate distance.

If such a public appointment were connected with the lectureship, every party would be benefited. The latter office would be filled by men of more knowledge and practical skill than those who now usually hold it. The public would have the advantage of the best evidence that could be procured, and, consequently, the greatest probability of a fair administration of the law. And the teacher would be enabled to give practical instruction to his pupils, instead of the present dry detail of cases and experiments. We can speak from experience when we say that the prosecution of one interesting investigation may leave a more valuable impression than a whole course of lectures. Such an appointment would of course be attended with a certain additional expense to the public; since it could not be expected that a man of standing in his profession should submit to the liability of being thus summoned from his regular employments without being handsomely remunerated. But whilst lawyers are receiving large incomes from the public purse, for aiding in the administration of justice, is it unreasonable to expect that, for such an important service to the public, medical men should receive a proper consideration for their services? The creation of such appointments—no sinecures, but well-remunerated offices—we regard as one of the means by which the state may encourage medical science with advantage to itself. In Scotland, where the sheriff has much more power than the coroner in England, considerable expense is often incurred for the purpose of the due administration of justice. We have known an eminent surgeon of Edinburgh sent for to Perth to tie the external iliac artery of a lad who had received a dangerous stab in the thigh. This was done by order of the sheriff, that no plea of *malum regimen* might possibly be set up in the event of the boy's death; as the blame might have been laid on the operation, had it been decided on and performed by a surgeon of less reputation.

If such a plan were pursued, the medical practitioner would be better able to concentrate his attention on the subjects more immediately within his province, and more important to his success. If he felt that he is not expected to conduct a minute chemical analysis, in a case of supposed poisoning, he would have more undivided thought to bestow on the observation of symptoms and post-mortem appearances; and he would study more closely the physiological action of poisons in the living system—an enquiry very closely connected with that which relates to the operation of medicinal agents or therapeutics. Still he should by no means be ignorant of the general principles upon which such an analysis is prosecuted; and he should be alive to all those occurrences which may affect its ultimate success. But it will scarcely be denied that he may be better occupied, as an intelligent practitioner, in attending to the medical history of the case, and in searching for the collateral evidence, of which *he* will, beyond all others, be most competent to indicate the sources, than in perplexing himself by endeavouring to apply his imperfect and half-forgotten chemical knowledge to an enquiry which may be more satisfactorily answered by the lecturer on forensic medicine in the nearest medical school.

From the foregoing remarks it will have been perceived that whilst we advocate the uniformity of medical *education*, we by no means object to a subdivision of medical *practice* into various departments. It is impossible for one individual to keep pace with the progress of knowledge on all branches of this extensive science; and, even if he were able to do so, it is highly improbable that he would be equally successful in the application of his knowledge to the treatment of the multifarious cases of disease and injury which present themselves in practice. It is universally allowed that the *division of labour* is favorable to perfection in all arts; and we do not see why it should be otherwise in the practice of the *healing art*, provided always that the choice of a special department be not made, until the *principles* of the science of medicine have been acquired. Here, again, our fundamental doctrine, of progress from the *general* to the *special*, comes into operation. We see no reason why any restraint should be placed upon a practitioner who, after a sound education, chooses to confine his attention to a very narrow class of subjects. Not only will the distinction between the surgeon, the physician, and the accoucheur always, from the very nature of things, be kept up wherever there is a population sufficient to maintain practitioners who, specially if not exclusively, profess these departments; but in all towns of large size or local importance, there will always be oculists and dentists, aurists and orthopœdists; and it seems to us essential to the improvement of our art that such should be the case. It requires a far greater share of experience than usually falls to the lot of any individual engaged in general practice, to make important additions to our knowledge of the pathology and treatment of diseases in the least degree uncommon. That so little has been hitherto done for science by those who have devoted themselves exclusively to restricted branches of practice, must be attributed to the very imperfect nature of their education. When the diseases of the ear and the teeth shall be as scientifically investigated and as competently treated as those of the eye are at present, we may look for important additions to our very defective knowledge of them.

The choice of a special department of practice, after the general principles of pathology and therapeutics have been acquired, very much resembles the choice of a special department of natural history, after a comprehensive survey has been taken of the whole organized creation. By none is it possible that an equally accurate and minute acquaintance with all groups of animated beings can be acquired within the compass of a single life. The two divisions of the world of organization—the animal and vegetable kingdoms—naturally suggest two separate objects of pursuit, zoology and botany. These, like medicine and surgery, may be advantageously pursued by separate individuals; but the fundamental principles of both sciences are (or ought to be) the same; and he who specially devotes himself to either will do so at a great disadvantage if not conversant with the general applications of those principles in the other. Again, zoology subdivides itself into as many subordinate branches of science as there are natural groups. We have the ornithologist and the conchologist, the herpetologist and the entomologist, the icthyologist and the actinologist. That such a subdivision tends to the perfection of the science cannot be doubted. But it is equally unquestionable that it would be far more advantageous if, before devoting himself to the pursuit of any one department, the young naturalist were to gain a general view of the whole science, and acquaint himself with the principal facts relating to the structure and actions of the chief subdivisions as well as of the animal kingdom. It can scarcely be conceived by those who have not attended to this subject, how many errors would have been avoided, how many new facts would have been observed, and how much perplexity to those who combine and generalize these facts would have been saved—in short with what vastly increased rapidity the science of natural history would have advanced if this course had been pursued by its votaries. At present we regard it as almost in its infancy. A vast amount of materials has been collected; but these are of so heterogeneous a nature as to admit only of very loose and unsatisfactory combination; and so they will remain until facts are observed in their relations to each other and to general principles, and not for their individual importance.

Just so it is with regard to medical science. We are firmly convinced that the minute subdivision of its applications to practice is favorable both to the success of the *arts* immediately founded upon it, and to the ultimate elevation of its own philosophical character. But it is essential to the accomplishment of this object that those who devote themselves to these subdivisions should have a thorough acquaintance with the principles which combine them all, and render them all subservient to the *philosophia medica* which is one and undivided. They are so many tributaries pouring the streamlets which originate from widely scattered sources into one noble river; but, that they should reach it, they must have *a right direction*, otherwise they become stagnated in inland lakes, and their waters serve not to swell the general current. We do not mean that an acquaintance with the *details* of each branch of practice is necessary or even desirable for him who restricts himself to one. Although it is interesting, it is not of vital importance to the physician to know whether a fractured thigh may be best treated by the straight splint or the double-inclined plane, or whether the circular amputation or the flap-operation be preferable, or what is the most advantageous point for tying the femoral artery in a case of popliteal aneurism; but he ought to know the principles upon which the treatment of fractures is conducted; the constitutional states which render an amputation desirable or contraindicate the performance of it; and the pathology and general treatment of aneurism. These, if he have studied them with attention, will remain in his mind, whilst the details which to him are practically unimportant are forgotten. Just so it will be in regard to all other subdivisions; even those of local affections, as the diseases of the eye, ear, and teeth. All these are connected with particular states of the constitution in such a manner that they can only be treated advantageously by one who has learned to discriminate these and apply his remedies to them; but it does not follow that an oculist should be a person to whose care we should like to commit a case of peritonitis, or that an aurist should be capable of extracting a stone from the bladder.

We are much mistaken if the view which we have now presented will not be found to reconcile all the principal difficulties of the subject. It has been contended on the part of some that, as pathology is but one science, and the fundamental principles of medicine and surgery are alike, there should be no division in the application; and that the constitutional origin of a great proportion of local diseases points out that they must all be put under the treatment of the same practitioners. On the other hand it has been urged that experience has shown the advantage of a subdivision; and that a degree of practical skill has been acquired by those who have concentrated their attention within a narrow circle of cases, which is not attainable by those who diffuse it over a wider range. Both doctrines are rightly founded; and we have shown how they may be harmonized.

It will be only, of course, in the midst of a large and dense population that the subdivisions we have alluded to can be advantageously practised. In country villages, as at present, the same individual must be physician and surgeon, oculist and dentist; and assistance of a higher character can only be obtained by those who can afford to obtain it from a distance. And, even in large towns, a great proportion of the practice will be in the hands of the same class, whose less expensive education and less ambitious establishments enable them to be satisfied with a more limited remuneration than is required by practitioners of a higher rank. But, whatever be their ultimate destination, we would have the same qualifications required in the first instance from all. We would have the same general acquaintance with the theory and practice of medicine and surgery made the condition of admission to practice; and we would have those who design to pursue some *special* department of their profession, first qualified as *general* practitioners, by such an education as the examination for the primary degree would require. Thus, in conformity with a great physiological principle, we should pass from the *general* to the *special;* and the higher grades would present, in the progress of their development, the permanent forms of the lower. Some of our readers may think such analogies forced and inappropriate; but we regard them as something more than superficial resemblances. They are the *common results* of the great principles of *philosophia prima* operating in different directions.

Higher degrees, both in medicine and surgery, should, we think, be attainable by those who seek for them, upon their producing evidence of more extended attainments in these departments, and of having been engaged for a certain time either in general practice or in attendance on hospitals. It seems to us that the two degrees should be distinct; since it is a useless tax upon the attention of him who intends to devote himself to the cure of internal diseases, to require the details of regional anatomy and operative surgery; and there are some departments of special pathology which need not be required from the pure surgeon. But there should be nothing to prevent the same individual from obtaining both diplomas, if he should deem it advisable.

We shall do no more, at present, than suggest the general outlines of a scheme by which our plan might be adapted to existing institutions, in England at least; provided always that the present governors of those institutions were willing to adopt it, and thus to avoid being altogether put aside. We would unite the Colleges of Physicians and Surgeons with the present University of London into one medical faculty; introducing some alterations into the constitution of all, so as to make them harmonize better with one another. To the University, we would commit the preliminary examination, and the examination for the first degree, that which qualifies for *general* practice. The College of Physicians should give the higher *medical* diploma; and the College of Surgeons the higher *surgical* diploma. The Apothecaries' Company should test the qualifications of dispensing chemists; and none but those who have obtained their license should be allowed to sell drugs by retail, or to make up prescriptions. We beg the unprejudiced attention of our readers to these suggestions.

